# A large abelisaurid (Dinosauria, Theropoda) from Morocco and comments on the Cenomanian theropods from North Africa

**DOI:** 10.7717/peerj.1754

**Published:** 2016-02-29

**Authors:** Alfio Alessandro Chiarenza, Andrea Cau

**Affiliations:** 1 Department of Earth Science and Engineering, Imperial College London, London, United Kingdom; 2 Earth, Life and Environmental Sciences, University of Bologna, Bologna, Italy; 3 Geological and Palaeontological Museum “G. Capellini,”Bologna, Italy

**Keywords:** Cenomanian, Morocco, Theropoda

## Abstract

We describe the partially preserved femur of a large-bodied theropod dinosaur from the Cenomanian “Kem Kem Compound Assemblage” (KKCA) of Morocco. The fossil is housed in the Museo Geologico e Paleontologico “Gaetano Giorgio Gemmellaro” in Palermo (Italy). The specimen is compared with the theropod fossil record from the KKCA and coeval assemblages from North Africa. The combination of a distally reclined head, a not prominent trochanteric shelf, distally placed lesser trochanter of stout, alariform shape, a stocky shaft with the fourth trochanter placed proximally, and rugose muscular insertion areas in the specimen distinguishes it from *Carcharodontosaurus*, *Deltadromeus* and *Spinosaurus* and supports referral to an abelisaurid. The estimated body size for the individual from which this femur was derived is comparable to *Carnotaurus* and *Ekrixinatosaurus* (up to 9 meters in length and 2 tons in body mass). This find confirms that abelisaurids had reached their largest body size in the “middle Cretaceous,” and that large abelisaurids coexisted with other giant theropods in Africa. We review the taxonomic status of the theropods from the Cenomanian of North Africa, and provisionally restrict the Linnean binomina *Carcharodontosaurus iguidensis* and *Spinosaurus aegyptiacus* to the type specimens. Based on comparisons among the theropod records from the Aptian-Cenomanian of South America and Africa, a partial explanation for the so-called “Stromer’s riddle” (namely, the coexistence of many large predatory dinosaurs in the “middle Cretaceous” record from North Africa) is offered in term of taphonomic artifacts among lineage records that were ecologically and environmentally non-overlapping. Although morphofunctional and stratigraphic evidence supports an ecological segregation between spinosaurids and the other lineages, the co-occurrence of abelisaurids and carcharodontosaurids, two groups showing several craniodental convergences that suggest direct resource competition, remains to be explained.

## Introduction

The dinosaurs from the Aptian-Cenomanian of North Africa are mainly known from a few articulated skeletons and several isolated bones, the majority of which are referred to medium- to large-sized theropod clades (i.e., Abelisauroidea, Carcharodontosauridae, Spinosauridae; Stromer, 1915; Stromer, 1931; Stromer, 1934; Russell, 1996; Sereno et al., 1996; Dal Sasso et al., 2005; Mahler, 2005; Brusatte & Sereno, 2007; Sereno & Brusatte, 2008; Smith et al., 2010; Cau, Dalla Vecchia & Fabbri, 2012; Cau, Dalla Vecchia & Fabbri, 2013; Ibrahim et al., 2014; Evers et al., 2015; Hendrickx, Mateus & Buffetaut, 2016). Whether the abundance of large theropods compared to other dinosaurs reflects a real ecological signal (i.e., an unusually unbalanced ecosystem; Läng et al., 2013) or a preservational, taphonomic or collecting biases (McGowan & Dyke, 2009) is still to be assessed. Here we describe an additional fossil specimen, adding further information on the known diversity of large-bodied African theropods. The fossil comes from the region of Taouz (Errachidia Province, Morocco, near the Moroccan-Algerian border) and was donated in 2005 to the Museo Geologico e Paleontologico “Gaetano Giorgio Gemmellaro” in Palermo (Italy) by a donor who had purchased it from a Moroccan fossil dealer. Many dinosaurian remains have been collected from the Tafilalt and Kem Kem regions (SE Morocco) by local inhabitants and fossil dealers and deposited in public institutions all over the world (McGowan & Dyke, 2009). As is usually the case (e.g. Evans et al., 2015; Cau, Dalla Vecchia & Fabbri, 2012; Hendrickx, Mateus & Buffetaut, 2016), this specimen was found by local people, and its exact horizon and locality is unknown. On the other hand, some information may be gleaned from the most recent and exhaustive review on the sedimentary geology of the Late Cretaceous North Africa dinosaur-rich units, also known as “Kem Kem Compound Assemblage” (KKCA *sensu* Cavin et al., 2010). These units are represented by the Ifezouane Formation and the overlying Aoufous Formation (Cavin et al., 2010), which are Cenomanian in age, and have been deposited along the south-western Tethyan margin before the late Cenomanian global marine transgression, represented in this region by the limestone unit of the Akrabou Formation (Cavin et al., 2010). The units included in the KKCA are the only dinosaur-bearing levels in the region of Taouz (Cavin et al., 2010). The matrix still encrusting the specimen (i.e., a consolidated red sandstone) closely recalls that present in other dinosaur fossils from the KKCA (e.g., Cau, Dalla Vecchia & Fabbri, 2013; Hendrickx, Mateus & Buffetaut, 2016; personal observations on material housed in the Natural History Museum in Milan; see Ibrahim et al., 2014). Based on its documented provenance and the lithological features mentioned above, we thus refer the fossil to the KKCA. In this study, we describe this specimen, compare it to other North African theropods, assess its phyletic relationships, and infer its body size.

## Abbreviations

KKCA, Kem Kem Compound Assemblage; OLPH, Olphin collection of the Museo Geologico e Paleontologico “Gaetano Giorgio Gemmellaro,” Università degli Studi di Palermo, Palermo, Sicily, Italy; NMC, Canadian Museum of Nature, formerly National Museum of Canada, Ottawa, Canada; ROM, Royal Ontario Museum, Toronto, Canada; SGM, Ministère de l’Énergie et des Mines, Rabat, Morocco.

## Systematic Palaeontology

Dinosauria Owen (1842).
Theropoda Marsh (1881).
Abelisauridae Bonaparte (1991).

### Locality and age

Based on the registry of the OLPH, the specimen was collected nearby the Moroccan-Algerian boundary just south of Taouz (Errachidia Province, Meknès−Tafilalet Region), Morocco. Following Cavin et al. (2010), the age of this fossil is considered as Late Cretaceous (Cenomanian).

### Material

**Figure 1:**
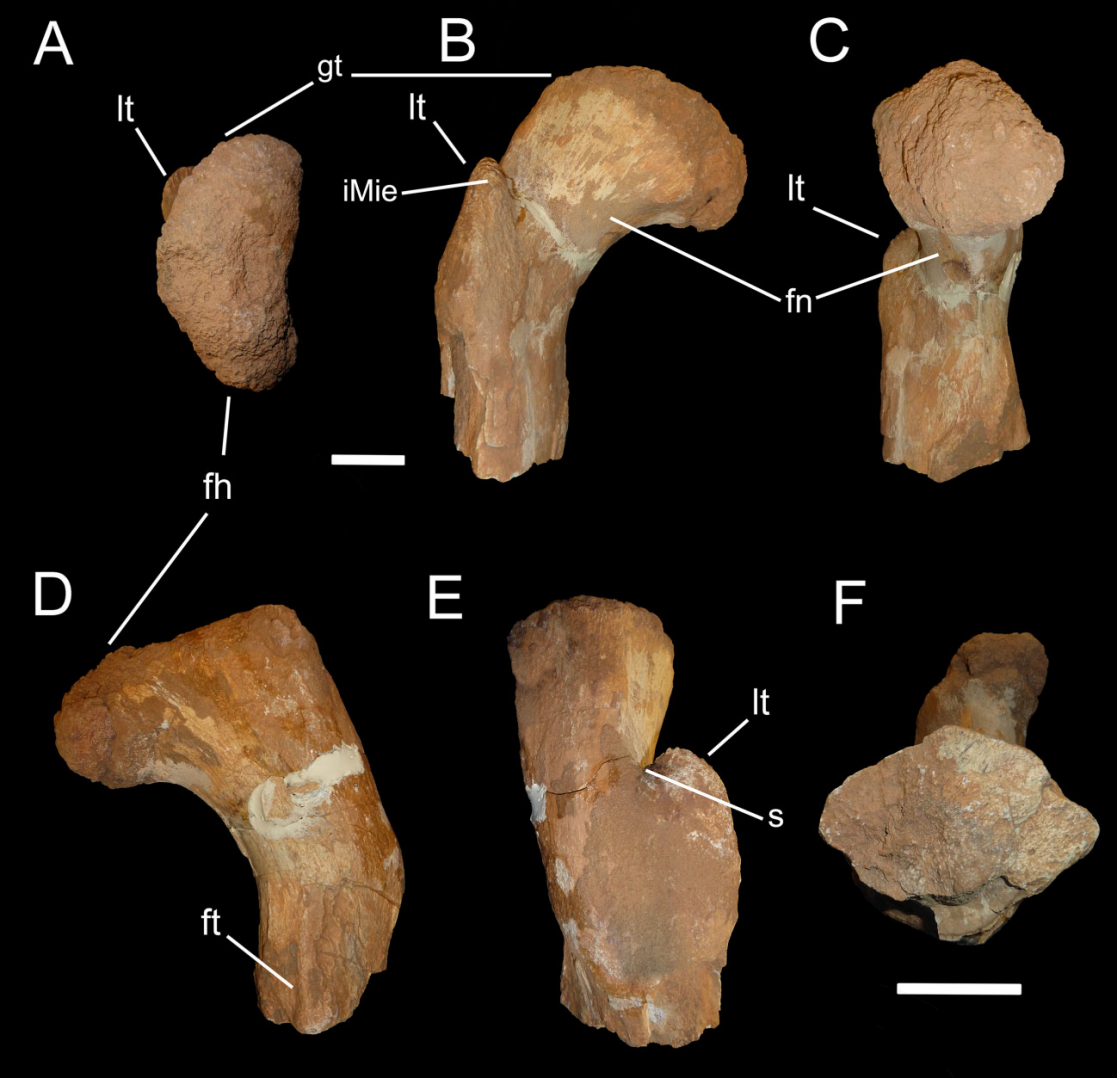
Abelisauridae indet. femur OLPH 025. (A) proximal view, (B) anterior view, (C) medial view, (D) posterior view, (E) lateral view, (F) distal view (not at same scale as other views). Scale bars, 5 cm. Abbreviations: gt, greater trochanter; iMie, insertion for the *M. iliofemoralis externus*; fn, femoral neck; s, shallow sulcus.

OLPH 025, partial proximal portion of a right femur (Fig. 1).

### Description and Comparison

Measurements for the specimen are included in Table 1. OLPH 025 is the proximal end of a femur, including the head, neck and trochanteric region. The preserved shaft is sigmoid in both anterior and posterior views (Fig. 1), as in *Berberosaurus liassicus* (Allain et al., 2007) and *Majungasaurus crenatissimus* (Carrano, 2007), and differs from the straighter shape in *Carcharodontosaurus saharicus* (Stromer, 1931). The femoral head (Fig. 1A) is anteroposteriorly compressed, subcircular in medial view (Fig. 1C), and has a narrow neck that curves anteriorly, placing the head anteromedially in proximal view, similar to the condition in *Carnotaurus*, *Ekrixinatosaurus*, *Rahiolisaurus*, *Xenotarsosaurus* and all other non-tetanuran theropods (Bonaparte, Novas & Coria, 1990; Novas et al., 2010). In anterior view (Fig. 1B), the dorsal margin of the femoral head is angled slightly distally rather than mainly perpendicular to the shaft, recalling *Masiakasaurus* and abelisaurids (Carrano, Sampson & Forster, 2002; Carrano, Wilson & Barrett, 2010; Carrano, 2007; Evans et al., 2015), whereas in *Carcharodontosaurus saharicus* and *Deltadromeus agilis* the head projects considerably proximally (Stromer, 1931; Evans et al., 2015). The lesser trochanter is broad anteroposteriorly and anteriorly projected, as in *Ceratosaurus, Masiakasaurus*, abelisaurids and basal tetanurans, set apart from the femoral head by a shallow sulcus as in *Ceratosaurus* and *Berberosaurus* and unlike the wide and deep cleft present in *Carcharodontosaurus saharicus* (Stromer, 1931). The lesser trochanter is positioned distally relative to the articular end, approaching proximally the level of the base of the head, differing from the more proximally placed trochanter present in *Deltadromeus* and most tetanurans (Madsen, 1976; Evans et al., 2015). The distal placement of the lesser trochanter is a plesiomorphic condition shared by coelophysoid-grade theropods (e.g., *Sarcosaurus*, Andrews, 1921), ceratosaurids (Madsen & Welles, 2000), and abelisauroids (Bonaparte, Novas & Coria, 1990; Le Loeuff & Buffetaut, 1991; Accarie et al., 1995; Martínez & Novas, 1997; Carrano, Sampson & Forster, 2002; Carrano, 2007). There is no evidence of a trochanteric shelf, although the posterolateral surface of the shaft at the level of the lesser trochanter appears damaged, so that any trace of even a faint trochanteric shelf (as in *Majungasaurus*; Carrano, 2007) may have been obliterated by erosion. Similar to *Berberosaurus* and *Majungasaurus* (Allain et al., 2007; Carrano, 2007), OLPH 025 does not show any evidence of the accessory trochanter, a feature widely present among neotetanuran theropods (Hutchinson, 2001) and illustrated on a femur referred to *Bahariasaurus* by Stromer (1934) and to *Deltadromeus* by Sereno et al. (1996). The anterior margin of the lesser trochanter bears a mound-like rugosity, interpreted as the insertion for the *M. iliofemoralis externus* (Hutchinson, 2001; Carrano, 2007). The distal (apical) and lateral surface of the lesser trochanter is extremely rugose, as in *Majungasaurus* (Carrano, 2007). In posterior view (Fig. 1D), toward the distal surface of the femur, a thin crista, proximodistally oriented, is set closer to the medial margin of the femur, extending gradually from the bone surface and oriented subparallel to the proximodistal axis of the diaphysis. This crest is interpreted as the proximal end of the ridge-like fourth trochanter. As in *Ceratosaurus* and abelisauroids (e.g., Madsen & Welles, 2000; Carrano, 2007), the fourth trochanter is placed more proximally than in tetanurans (e.g., *Allosaurus*, Madsen, 1976, plate 50). The fourth trochanter is more medially than centrally set along the posterior surface, as in *Ceratosaurus* (Madsen & Welles, 2000). In proximal view, the femur head appears “kidney-shaped” with the lesser trochanter barely visible on the anteromedial corner, differing from the condition in tetanurans and noasaurids, where the lesser trochanter is more widely exposed in proximal view (e.g., *Allosaurus fragilis*, personal observations; *Masiakasaurus*, Carrano, Sampson & Forster, 2002). In distal view (Fig. 1F), the femoral shaft is slightly more anteroposteriorly compressed, with an approximately triangular to rhomboidal outline in section at the level of the fourth trochanter, and with the apex pointing anteriorly, as in *Ceratosaurus, Masiakasaurus,* and abelisaurids (Madsen & Welles, 2000; Carrano, 2007; Carrano, Loewen & Sertich, 2011). This shape differs from the more rounded cross-section of tetanuran femora (e.g., Madsen, 1976, fig. 24B). As in the vast majority of theropods, but differing from a KKCA femur referred to *Spinosaurus* by Ibrahim et al. (2014), the medullary cavity is large (using the better preserved anteromedial quarter of the section, the radius of the medullary cavity is about half the length of both principal section axes).

**Table 1:** Selected measurements (in mm) of OLPH 025.

Proximal surface, anteroposterior length from acetabular surface to greater trochanter	170
Proximal surface, minimum transverse width at mid-length	100
Anterior view, maximum proximodistal length of preserved bone	330
Anterior view, proximodistal depth of articular surface	95
Head, articular surface anteroposterior diameter	150
Greater trochanter, maximum anteroposterior diameter	90
Greater trochanter, proximodistal depth above lesser trochanter base	105
Shaft, preserved distal surface, anteroposterior diameter vs preserved width	120 × 90

## Results and Discussion

### Taxonomy and inclusiveness of the KKCA theropod taxa

**Figure 2:**
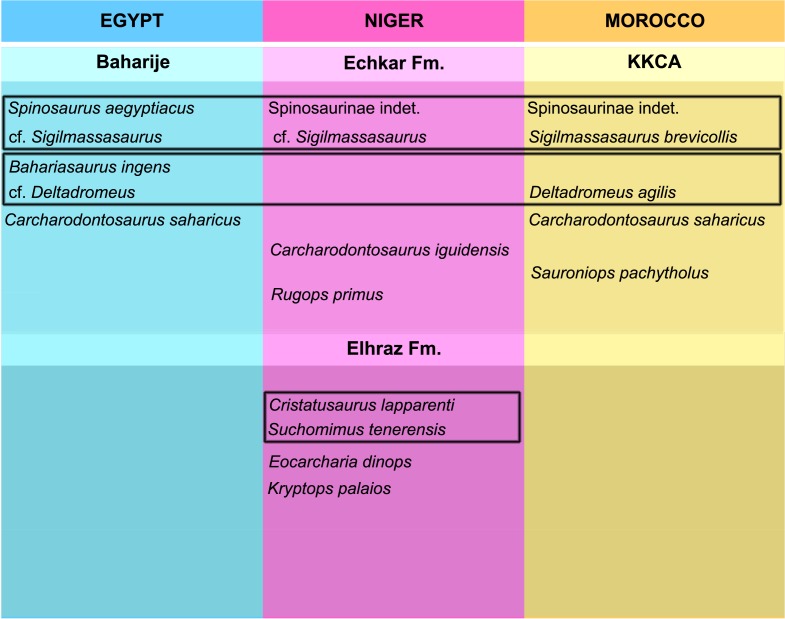
Main theropod faunal assemblages from the Aptian-Cenomanian of North Africa. Taxa enclosed in rectangles have been considered as synonyms by some authors and distinct by others (see Russell, 1996; Sereno, Wilson & Conrad, 2004; Cau, Dalla Vecchia & Fabbri, 2013; Ibrahim et al., 2014; Evers et al., 2015).

Most African theropod taxa are based on isolated material, often single bones, or include referred material that in many cases lacks overlapping elements with the type specimens (e.g., *Kryptops palaios*, Sereno & Brusatte, 2008; see discussion in Carrano, Benson & Sampson (2012); *Eocarcharia dinops*, Sereno & Brusatte, 2008). Since referral of isolated and non-overlapping specimens to the same taxon is a hypothesis itself, we briefly review here the taxonomic status of the known theropod taxa from the KKCA and–where relevant to the discussion–from penecontemporaneous assemblages from North Africa (Fig. 2).

### Carcharodontosauridae

**Figure 3:**
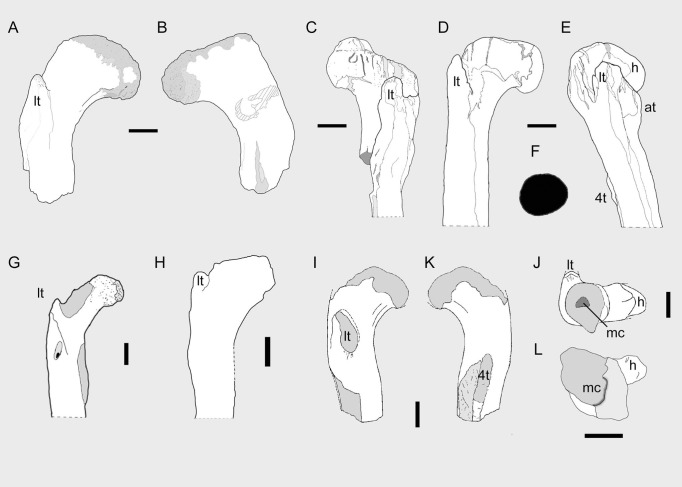
Theropod femora from the Cenomanian of Egypt and the ‘Kem Kem Compound Assemblage’. OLPH 025 in anterior (A) posterior (B) and distal (L) views; scale bar 5 cm. (C) *Carcharodontosaurus saharicus* femur in anterolateral view (re-drawn from Stromer, 1931, table I), scale bar, 5 cm. (D) and (E) cf. *Baharisaurus ingens* femur, referred to *Deltadromeus* by Sereno et al. (1996), re-drawn from Stromer (1934, table III) in anterior (D) and lateral (E) views; scale bar, 5 cm. (G) left femur of a theropod (ROM 64666, reversed from right) referred to *Deltadromeus agilis* by Evans et al. (2015), in anterior view; scale bar, 1 cm. (H) left type femur of *Deltadromeus agilis* (SGM Din-2, reversed from right) in posterior view; scale bar, 5 cm. NMC 41869, a right femur referred to Russell (1996) to Theropoda indet. (“bone taxon M”) in (I) anterior, (J) distal, (K) posterior views; scale bar, 5 cm.

The original types of “*Megalosaurus saharicus*” were two isolated teeth from the Late Cretaceous “Continental Intercalaire” units of Algeria (Depéret & Savornin, 1925; see discussion by Brusatte & Sereno (2007)). The genoholotype of *Carcharodontosaurus* is based on a partial skeleton from the Baharjie assemblage of Egypt that includes teeth comparable to those of “*M. saharicus*,” and, among other elements, a well-preserved femur (Stromer, 1931; Fig. 3C). That material (and all other theropod bones described by Stromer (1915), Stromer (1931) and Stromer (1934)) was destroyed during World War II. Brusatte & Sereno (2007) designated a partial skull from the Cenomanian of the KKCA (see Sereno et al., 1996) as the neotype of *Carcharodontosaurus saharicus*. This material lacks a femur, preventing direct comparison with the Palermo specimen. Although in overall morphology the neotype of *C. saharicus* (Sereno et al., 1996; Brusatte & Sereno, 2007) closely matches the overlapping cranial material of the destroyed Egyptian specimen (Stromer, 1931), the two specimens differ in the shape of the maxillary interdental plates, that are quadrangular in medial view and apically flattened in the Moroccan specimen (Brusatte & Sereno, 2007, fig. 2; Hendrickx & Mateus, 2014, supplementary information), whereas are depicted as subtriangular in medial view and apically pointed in the Egyptian specimen (Stromer, 1934, plate 1, fig. 6a). This difference may be taxonomically significant because it also differentiates the holotype of *Carcharodontosaurus iguidensis* from the neotype of *Carcharodontosaurus saharicus* (Brusatte & Sereno, 2007, fig. 2), and is a phylogenetically informative feature among theropod species (see Hendrickx & Mateus, 2014, supplementary information). The type material of *Carcharodontosaurus iguidensis* includes an isolated maxilla from the Echkar Formation of Niger (Brusatte & Sereno, 2007). The referred material (partial skull and vertebrae) was discovered three kilometers away from the type maxilla and lacks overlapping elements with the latter (Brusatte & Sereno, 2007). Brusatte & Sereno (2007: 905) referred isolated bones from the Echkar Formation to *C. iguidensis* because they “closely match the morphology of *C. saharicus* and because it is unlikely that there would be more than three contemporaneous large-bodied carnivores in the same formation (*Rugops primus*, *Spinosaurus* sp., *Carcharodontosaurus iguidensis*).” We see no reason why the number of large-bodied carnivores in a geological formation should be limited to three, or to refer all carcharodontosaurid specimens from the same formation to a single species when no overlapping material is available (see Cau, Dalla Vecchia & Fabbri, 2013 and reference therein). This raises doubts about the referral of that material to *C. iguidensis*. In particular, the referred material of *C. iguidensis* includes vertebrae referable to the spinosaurid *Sigilmassasaurus* or a closely related taxon (McFeeters et al., 2013; Evers et al., 2015), indicating that it represents a multitaxic association. Among the material referred to *C. iguidensis*, a dentary and braincase were discovered in situ embedded in sandstone of the Echkar Formation and closely associated in a small area (Brusatte & Sereno, 2007), supporting their referral to a single individual. This material shares synapomorphies of Carcharodontosauridae (Brusatte & Sereno, 2007) but lacks synapomorphies of the subclade Carcharodontosaurinae present in both *Carcharodontosaurus* and *Giganotosaurus* (Coria & Currie, 2002): the thickened lacrimal facet of frontal, the invaginated anteromedial corner of the supratemporal fossa, and the exit of the trigeminal foramen posterior to the nuchal crest.

As stated by Brusatte & Sereno (2007), the braincase referred to *C. iguidensis* shows the facet for contact with the prefrontal-lacrimal on the frontal is shallower than in both *C. saharicus* and *Giganotosaurus* (note that, in derived carcharodontosaurids, the prefrontal is reduced and fused to the lacrimal; therefore, the lacrimal facet of frontal in carcharodontosaurids is homologous to the prefrontal facet of basal allosauroids, Sereno & Brusatte (2008)). This feature was listed by Brusatte & Sereno (2007) among the three features differentiating the frontal of *C. iguidensis* from that of *C. saharicus*, “the latter probably exhibiting the derived condition” (Brusatte & Sereno, 2007: 907). Thus, Brusatte & Sereno (2007) implicitly noted that *C. iguidensis* shows the plesiomorphic condition compared to *C. saharicus*. In particular, the lacrimal facet in the neotype frontal of *C. saharicus* is 65 mm deep, about 40% the length of the frontal (a bone stated by Brusatte & Sereno (2007), to be identical in length to the 150 mm long frontal of *C. iguidensis*). In the frontal referred to *C. iguidensis*, the same facet is reported to be 35 mm deep (Brusatte & Sereno, 2007: 908), about 23% the length of the bone. Coria & Currie (2002) reported that on the 200 mm long frontal of *Giganotosaurus carolinii* holotype, the prefrontal [-lacrimal] facet is 67.5 mm deep, about 33% the length of the frontal. In the braincase of *Acrocanthosaurus atokensis* described by Eddy & Clarke (2011), the depth of the prefrontal facet of the frontal is about 40–45 mm deep (Eddy & Clarke, 2011, fig. 12), about 23% the length of the frontal (Eddy & Clarke, 2011, table 1). Note that the latter is the same value as for the frontal referred to *C. iguidensis*. In *Shaochilong maortuensis*, the depth of the same facet is 25% the length of the frontal (based on measurements provided by Brusatte et al. (2010)). In more basal allosauroids, the depth of the prefrontal facet of frontal is about 20–25% the length of the bone (e.g., *Sinraptor dongi*, see Currie & Zhao, 1994, figs. 7B–7D). Therefore, *C. saharicus* and *Giganotosaurus* share a prefrontal-lacrimal facet that is more than 30% the length of the frontal, and this derived feature may represent a synapomorphy of Carcharodontosaurinae absent in the frontals of other allosauroids, including that referred to *C. iguidensis.* Although a deep lacrimal facet of frontal is present also in *Sauroniops* (Cau, Dalla Vecchia & Fabbri, 2012; Cau, Dalla Vecchia & Fabbri, 2013), this feature is probably not homologous to the condition in other carcharodontosaurids because in the latter the facet is thickest in its posterior margin, not along its anterior margin, as in *Eocarcharia* and *Sauroniops* (Sereno & Brusatte, 2008; Cau, Dalla Vecchia & Fabbri, 2013).

Furthermore, Brusatte & Sereno (2007) reported that “the anteromedial corner of the supratemporal fossa is deeply invaginated in *C. saharicus*, but forms a near vertical, broadly arched wall in [the braincase referred to] *C. iguidensis*” (Brusatte & Sereno, 2007: 908). *Carcharodontosaurus saharicus* shows the derived condition, which is due to the extensive development of a medial shelf overlapping the anteromedial corner of the supratemporal fossa (Coria & Currie, 2002). The latter feature is only shared by *Giganotosaurus carolinii* among allosauroids (Coria & Currie, 2002), including other carcharodontosaurids (Sereno & Brusatte, 2008; Brusatte et al., 2010; Eddy & Clarke, 2011), and is thus interpreted as a synapomorphy of Carcharodontosaurinae. Although a medial shelf is incipiently developed in other carcharodontosaurids (e.g., *Acrocanthosaurus*, Coria & Currie, 2002), only *C. saharicus* and *Giganotosaurus* show a deeply invaginated anteromedial corner of the supratemporal fossa due to the extreme development of the shelves. The absence of the invaginated anteromedial corner of the supratemporal fossa on the braincase from Niger is an additional feature challenging its referral to a species of Carcharodontosaurinae.

In their phylogenetic analysis of Allosauroidea, Brusatte & Sereno (2008) used the position of the trigeminal foramen exit in the braincase relative to the nuchal crest as a phylogenetically informative character, and defined the states as: “braincase, trigeminal (nerve V) foramen, location relative to nuchal crest: anterior or ventral (0); posterior (1) (Brusatte & Sereno, 2008: 26).” According to this character statement, the braincase referred to *C. iguidensis* should be scored as “0,” as its trigeminal foramen is reported to be ventral to the nuchal crest (Brusatte & Sereno, 2007: 910), as in *Sinraptor*, and not “1,” as in *C. saharicus*, *Giganotosaurus* and *Shaochilong* (Brusatte et al., 2010), the latter three showing a more posteriorly placed foramen. Therefore, according to Brusatte & Sereno (2008), the position of the trigeminal foramen in the braincase referred to *C. iguidensis* is plesiomorphic relative to the conditions in both *C. saharicus* and *Giganotosaurus*, further challenging the referral of that specimen (regardless to the placement of the taxa *C. iguidensis*, based on the type maxilla, and *Shaochilong*) to Carcharodontosaurinae.

Therefore, the braincase referred to *C. iguidensis* shows a combination of features intermediate between carcharodontosaurine (e.g., Coria & Currie, 2002) and non-carcharodontosaurine (e.g., Eddy & Clarke, 2011) carcharodontosaurids. Some of these features were considered by Brusatte & Sereno (2007) autapomorphies of *C. iguidensis*, and thus, accepting the referral of the braincase to the latter taxon, should be considered reversals to the non-carcharodontosaurine (plesiomorphic) condition. We cannot dismiss that some of these differences between the Nigerine braincase and the carcharodontosaurines are ontogenetic in nature (implying that the braincase described by Brusatte & Sereno (2007), pertains to an individual ontogenetically less mature than the Moroccan neotype of *C. saharicus*). Nevertheless, assuming that the material belongs to a mature individual (Brusatte & Sereno, 2007), this plesiomorphic combination of features challenges the referral of the braincase from Niger to *Carcharodontosaurus*. Brusatte & Sereno (2007) listed the presence of large internal carotid foramina and deep paracondylar pneumatic foramina and the presence of a deep basisphenoid fossa as diagnostic features of *Carcharodontosaurus*, supporting the referral of the Nigerine braincase to the latter genus. Nevertheless, this combination of features is also shared by *Giganotosaurus* (Coria & Currie, 2002), indicating that they are synapomorphies of a clade more inclusive than *Carcharodontosaurus* and thus not diagnostic for the latter genus alone. In North African fossil assemblages, it is not uncommon to have two similarly-sized and closely related theropod taxa occurring in the same unit (e.g., Stromer, 1934; Cau, Dalla Vecchia & Fabbri, 2012; Cau, Dalla Vecchia & Fabbri, 2013; Fanti et al., 2014; Evers et al., 2015; Hendrickx, Mateus & Buffetaut, 2016). Therefore, in the absence of overlapping material with the type of *C. iguidensis* (i.e., maxillae), and lacking unambiguous braincase autapomorphies of *Carcharodontosaurus*, we cannot exclude that the associated dentary-braincase material pertains to a carcharodontosaurid species distinct from (and more basal than) *C. iguidensis*. Alternatively, if the referral of that material to *C. iguidensis* is confirmed, its combination of features may support a more basal placement for the latter taxon relative to Carcharodontosaurinae. In conclusion, in order to avoid the introduction of a possible chimera (in particular, in phylogenetic analyses), we provisionally exclude the referred material from *C. iguidensis*, restricting the latter name to the type maxilla.

*Sauroniops pachytholus* is based on a large, isolated frontal from the KKCA (Cau, Dalla Vecchia & Fabbri, 2012; Cau, Dalla Vecchia & Fabbri, 2013). The specimen differs from all other known theropod frontals from the “mid-Cretaceous” of North Africa, in particular *Carcharodontosaurus saharicus* and the braincase referred to *C. iguidensis* (Sereno et al., 1996; Brusatte & Sereno, 2007; Sereno & Brusatte, 2008), and shares a set of unique features with the frontals from the Aptian of Niger referred to the basal carcharodontosaurid *Eocarcharia dinops* (Brusatte & Sereno, 2008).

### Spinosauridae

*Sigilmassasaurus brevicollis* is based on isolated presacral vertebrae from the KKCA (Russell, 1996) and was recently rediagnosed by McFeeters et al. (2013) and Evers et al. (2015), including material that was referred to *Spinosaurus* (as *Sp. maroccanus*) by Russell (1996). Ibrahim et al. (2014) suggested the referral of several specimens from the Cenomanian of Morocco to *Spinosaurus aegyptiacus*, including the material previously referred to *Sigilmassasaurus* (McFeeters et al., 2013). This hypothesis was recently challenged by Evers et al. (2015), who referred part of the material of *Spinosaurus* (*sensu* Ibrahim et al., 2014) to *Sigilmassasaurus*, the latter considered a distinct spinosaurid taxon. Evers et al. (2015) and Hendrickx, Mateus & Buffetaut (2016) provided evidence for the presence of more than one spinosaurid taxon in the KKCA. Accordingly, in this study, we distinguish between the material introduced by Ibrahim et al. (2014) and the material of *Sigilmassasaurus* (*sensu* Evers et al., 2015), and provisionally restrict the name *Spinosaurus aegyptiacus* to the now lost holotype from Egypt, described by Stromer (1915). We agree with Evers et al. (2015) that the erection of a neotype for *S. aegyptiacus* based on the material from Morocco described by Ibrahim et al. (2014) is not adequately justified. It should be noted that Evers et al. (2015) have rediagnosed *Si. brevicollis* based on comparison with the known presacral vertebrae of Spinosauridae, and listed a set of characters that does not completely overlap with that used by Russell (1996). Accordingly, the taxon *Sigilmassasaurus* (sensu Evers et al., 2015) is less inclusive than *Sigilmassasaurus* (sensu Russell, 1996) because some of the diagnostic features of the latter are now known to be shared by other spinosaurid taxa (e.g., *Baryonyx*, *Ichthyovenator*; see Evers et al., 2015). Therefore, we cannot dismiss that some “*Sigilmassasaurus*-like” vertebrae from the KKCA, referred to *Sigilmassasaurus* by Russell (1996), may eventually prove to not belong to *Sigilmassasaurus* (sensu Evers et al., 2015) but to other coeval spinosaurids, such as *Spinosaurus* (see Ibrahim et al., 2014; Hendrickx, Mateus & Buffetaut, 2016). Furthermore, we note that, following the distinction between *Spinosaurus* and *Sigilmassasaurus* proposed by Evers et al. (2015) and Hendrickx, Mateus & Buffetaut (2016), the large and well-preserved spinosaurid snout from the KKCA described by Dal Sasso et al. (2005) cannot be referred unambiguously to the former taxon rather than the latter (see also the lack of resolution among spinosaurid taxa in the phylogenetic topology of Evers et al. (2015)). It is worth noting that Milner (2001) described a large spinosaurid dentary from the KKCA, comparable in length to the type dentary of Stromer (1915), that differs from the latter in the overall stouter proportion of the bone, in the shape of the alveolar margin, and in the number and placement of the alveoli (at least 17, compared to 15 in the Egyptian specimen). This find further supports the hypothesis that the Moroccan material includes at least one spinosaurine taxon distinct from the Egyptian species. Since a discussion of the inclusiveness of the name *Spinosaurus aegyptiacus* (Ibrahim et al., 2014; Evers et al., 2015; Hendrickx, Mateus & Buffetaut, 2016) is beyond the aims of this study, and pending a taxonomic revision of the spinosaurid material from the Cenomanian of Morocco (in particular, the material introduced by Ibrahim et al. (2014), (Maganuco, 2014, personal communication), (N. Ibrahim, personal communication in Hendrickx, Mateus & Buffetaut (2016)), we suggest to refer the KKCA material that cannot be referred unambiguously to either *Spinosaurus* or *Sigilmassasaurus* to Spinosaurinae indet., the least inclusive taxonomic unit all authors agree that material belongs to Hendrickx, Mateus & Buffetaut (2016).

Russell (1996) described the partial femur of an indeterminate theropod (“bone taxon M”), characterized by a robust shaft, declined head, distally placed lesser trochanter, and hypertrophied fourth trochanter. Carrano & Sampson (2008) noted the overall similarities to femora of basal theropods, including abelisaurids. As outlined below, based on presence of unique features of the femur referred to *Spinosaurus* by Ibrahim et al. (2014), we refer “bone taxon M” to Spinosauridae.

### Ceratosauria

*Deltadromeus agilis* is based on a single, partial skeleton from the KKCA (Sereno et al., 1996) including the femora, the latter showing autapomorphic features. Originally interpreted as a coelurosaur (Sereno et al., 1996), more recent phylogenetic analyses agree in placing it among Ceratosauria (e.g., Sereno, Wilson & Conrad, 2004; Carrano & Sampson, 2008; Cau, Dalla Vecchia & Fabbri, 2012). Sereno et al. (1996, note 32) distinguished *D. agilis* from *Bahariasaurus ingens* (from penecontemporary levels of Egypt, Stromer, 1934) on the basis of three features in the pubis and ischium, and referred part of the Egyptian material, that was first referred to *Bahariasaurus* by Stromer (1934), to the Moroccan taxon. This interpretation was challenged by Carrano & Sampson (2008), who suggested (without providing justification) that the bone interpreted by Sereno et al. (1996) as the distal end of the pubis of the holotype of *Deltadromeus agilis* may pertain to the ischium, thus invalidating the differences from the type material of *Bahariasaurus ingens*. The majority of the elements referred alternatively to *Bahariasaurus* or *Deltadromeus* share basal ceratosaurian and abelisauroid synapomorphies (Carrano & Sampson, 2008), including elongate, rectangular anterior caudal neural spines, dorsoventrally expanded acromion and coracoid, gracile and straight humerus with reduced deltopectoral crest, triangular obturator flanges on pubis and/or ischium, expanded ischial foot, prominent muscular insertions on laterodistal margin of femur, large fossa on proximomedial surface of fibula bounded posteriorly by a lip, and gracile fourth metatarsal with reduced distal end (Janensch, 1925; Stromer, 1934; Sereno et al., 1996; Carrano, Sampson & Forster, 2002; Carrano & Sampson, 2008; Novas et al., 2008). Therefore, even if not synonymous, the two taxa may be related to noasaurids or form a clade of mid- to large-bodied and gracile-limbed basal ceratosaurians, including *Limusaurus* and *Elaphrosaurus* (Carrano & Sampson, 2008; Cau, Dalla Vecchia & Fabbri, 2013), for which the name Bahariasauridae (Huene, 1948) is available. Additional information on the femoral morphology of *Deltadromeus* was recently provided by Evans et al. (2015). A large theropod femur from the Cenomanian of Egypt was assigned by Stromer (1934: 36, pl. 3, fig. 5) to *Bahariasaurus* (Figs. 3D and 3E). Nevertheless, the type material of *B. ingens* lacks femora (Stromer, 1934); therefore, no direct evidence for referring the former specimen to that species is available. Sereno et al. (1996) referred that femur to *Deltadromeus* (Figs. 3G and 3H), based on their resemblance to the Moroccan material and shared presence of autapomorphies of the latter (Sereno et al., 1996, note 5). Although this referral may further support a close relationship (if not synonymy) between *Deltadromeus* and an Egyptian gracile-limbed theropod (that may be *Bahariasaurus* itself), the Egyptian femur differs from the published holotype femur of *D. agilis* because it appears proportionally stouter, lacks a proximally directed head, and shows a proximodistally shorter lateral accessory crest on the distal end (Stromer, 1934; Evans et al., 2015). Some of these differences, in particular the stouter overall proportions, may be size-related because the Egyptian specimen is about one time and a half larger than the Moroccan specimen. Other differences are more difficult to explain as due to ontogenetic change. In particular, the Egyptian specimen (Stromer, 1934, Table III, fig. 5a; Rauhut, 1995, fig. 5F) shows a neck that is not particularly inclined proximally compared to *Deltadromeus* (see Evans et al., 2015, fig. 3B). Since the proximal inclination of the femoral neck is a weight-bearing adaptation shared by several large-bodied dinosaurs (Rauhut, 1995; Carrano, 1998), the absence of this feature in the more massive Egyptian specimen compared to the more gracile Moroccan specimen is unexpected if we assume that the two femora belong to the same ontogenetic trajectory, and raises question for the referral of the former to the same species of the latter.

Among the isolated bones from the KKCA described by Russell (1996), one posterior dorsal vertebra (“bone taxon C”) was referred by the latter author to a large-bodied taxon distinct from *Carcharodontosaurus*, *Sigilmassasaurus* and *Spinosaurus* due to its unique combination of features. Among them, the vertebra is unusual in the relatively large size of the neural canal and the shape of the latter, described as dorsally separated into two halves by a low longitudinal ridge extending along the neural canal roof, and ventrally incised deeply into the centrum (Russell, 1996: 378). Both the large size and “heart-like” outline of the neural canal are shared by the posterior dorsal vertebra of a fragmentary theropod from the Lower Cretaceous of Libya (Smith et al., 2010), suggesting a possible relationship between these taxa. The Libyan taxon is referred to a large-bodied (estimated body length: 7–9 m, Smith et al., 2010, table 1) and gracile-limbed ceratosaurian based on the morphology of the femur and tibia and shows a unique combination of features that supports its referral to a new taxon (Smith et al., 2010).

Several isolated bone elements from the KKCA have been referred to Abelisauridae (Russell, 1996; Mahler, 2005; Carrano & Sampson, 2008; D’Orazi Porchetti et al., 2011). One abelisaurid, *Rugops primus*, is present in penecontemporary levels from Niger (Sereno, Wilson & Conrad, 2004). It is noteworthy that no abelisaurid material is known from the Baharjie assemblage (Stromer, 1931; Stromer, 1934; Carrano & Sampson, 2008), whereas the same clade is reported in the majority of North African “middle” Cretaceous localities (e.g., Sereno, Wilson & Conrad, 2004; Sereno & Brusatte, 2008; Fanti et al., 2014). Carrano & Sampson (2008) questioned the referral of the isolated maxillary fragment from the KKCA described by Mahler (2005) to Abelisauridae, noting that most of the features discussed by the latter author are shared by carcharodontosaurids. Nevertheless, additional abelisaurid synapomorphies, differentiating it from carcharodontosaurids, are present in this specimen (Cau & Maganuco, 2009).

### Problematic material from the KKCA referred to Theropoda

McFeeters (2013) reviewed the record of small-sized bones of theropods from the KKCA, concluding that most of the elements cannot be unambiguously referred to small-bodied taxa rather than immature individuals of large-bodied species. Among these elements, Riff et al. (2004) referred a small dorsal vertebra to Paraves, noting overall similarities with *Rahonavis*. Nevertheless, the specimen lacks unambiguous paravian or avialan synapomorphies. In particular, the large size of the neural canal, considered by Riff et al. (2004) as an avian synapomorphy, is a size-related feature homoplastically present among all small-bodied theropods (including small abelisauroids; see Carrano, Sampson & Forster, 2002) and also non-theropod taxa (e.g., crocodyliforms; see Lio et al., 2012).

Cau & Maganuco (2009) referred an isolated distal caudal vertebra from the KKCA to a new mid-sized theropod, that they named *Kemkemia auditorei*. Most of the unique features (among theropods) present in this specimen are shared by crocodyliforms, challenging the referral of that vertebra to Theropoda (Lio et al., 2012). Among the unique features of *K. auditorei*, the robust (mediolaterally thick) neural spine with a concave dorsal surface is currently unreported among crocodyliform distal caudal vertebrae (Lio et al., 2012) and may represent an autapomorphic feature of this taxon. Although unreported among crocodyliforms, the unusual mediolateral broadening of the neural spine of *K. auditorei* is shared by a series of isolated caudal vertebrae from the KKCA referred to either *Sigilmassasaurus* by Russell (1996, figs. 12F–12G) or to an indeterminate dinosaur by McFeeters et al. (2013, fig. 10), and, most recently, to *Spinosaurus* by Ibrahim et al. (2014). Stromer (1934) described a similar caudal vertebral morphotype among the material of “*Spinosaurus* B” (Russell, 1996; Ibrahim et al., 2014; Evers et al., 2015). It is noteworthy that the Egyptian vertebra illustrated by Stromer (1934) differs from the Moroccan vertebrae of Russell (1996; see also McFeeters et al., 2013, fig. 10) in the unusual transversal broadening of the neural spine, the latter showing lateral margins that diverge apically in anterior view (in the Moroccan material, the lateral margins of the neural spine are subparallel in anterior view, Russell, 1996; McFeeters et al., 2013, fig. 10W). It is unclear whether this difference among the Moroccan and Egyptian vertebrae is merely positional, taxonomically significant, or–as suggested by Russell (1996)–a pathological feature of the Egyptian specimen. The holotype of *K. auditorei* also shares with the KKCA caudal vertebrae described by Russell (1996) the absence of a ventral sulcus in the centrum, the marked reduction of the zygapophyses, and the combination of a well-developed neural spine even in distal vertebrae lacking the ribs; whereas it differs from them in the presence of pre- and postspinal laminae (Cau & Maganuco, 2009; McFeeters et al., 2013). All the known caudal vertebrae referred to *Sigilmassasaurus* and/or *Spinosaurus* pertain to the proximal and middle parts of the tail and thus cannot be compared directly with the more-distally placed holotype of *K. auditorei* (Cau & Maganuco, 2009). Given the series of morphological convergences between spinosaurines and crocodyliforms (Ibrahim et al., 2014), the combination of crocodyliform-like and “*Sigilmassasaurus*-like” features in *Kemkemia* is intriguing: therefore, it is currently unclear whether the holotype of *K. auditorei* is referable to a crocodyliform or a spinosaurid.

### Affinities of OLPH 025

The combination of large size, presence of both lesser trochanter and large medullary cavity in the shaft unambiguously indicates that OLPH 025 belongs to a theropod dinosaur (Sereno, 1999). Russell (1996) described the proximal portion of a femur from the ‘Kem Kem beds’ of Morocco (NMC 41869; Figs. 3I–3J) and referred it to an indeterminate theropod. OLPH 025 differs from NMC 41869 in having a larger medullary cavity, a more reclined head that is directed anteromedially, and in the presence of a distinct anterior corner of the shaft in distal view (Russell, 1996, figs. 25A–25C). Based on Russell (1996, fig. 25C), NMC 41869 shows the head that is directed perpendicular to the anteroposterior axis of the shaft (indicated by the placement of the lesser and fourth trochanters), thus medially directed as in tetanurans and not anteromedially as in abelisauroids and OLPH 025. Russell (1996) described the fourth trochanter of NMC 41869 as “heavily developed.” Furthermore, the cross-section of the shaft depicted by Russell (1996, fig. 25C) shows a smaller medullary cavity than OLPH 025. Since the latter two features are reported exclusively in *Spinosaurus* (*sensu* Ibrahim et al., 2014) among large-bodied theropods, we refer NMC 41859 to Spinosauridae. In overall features, OLPH 025 is more robust than a theropod femur from the Cenomanian of Egypt assigned by Stromer (1934: 36, pl. 3, fig. 5) to *Bahariasaurus*. Similarly to NMC 41869, the lesser trochanter of OLPH 025 lies more distally relative to the femoral head, a condition that differs from cf. *Bahariasaurus* and *Carcharodontosaurus* (Stromer, 1931, pl. 1, fig. 14). Furthermore, OLPH 025 differs from the large femur referred to *Bahariasaurus* by Stromer (1934) in the more distally placed lesser trochanter and the absence of a distinct accessory trochanter. OLPH 025 differs from *Deltadromeus* in the more reclined (distally directed) projection of the head, in the more distal placement of the lesser trochanter, and in the overall stouter proportions of the bone (Evans et al., 2015).

The other large-bodied theropods based on isolated material from the KKCA (i.e., *Sauroniops pachytholus* and *Sigilmassasaurus brevicollis*) cannot be directly compared to the Palermo specimen since no femora are known for either taxon. Both *Sauroniops* and *Sigilmassasaurus* are interpreted as tetanurans (i.e., respectively, a carcharodontosaurid and a spinosaurid, possibly synonymous with *Spinosaurus*; Cau, Dalla Vecchia & Fabbri, 2012; Cau, Dalla Vecchia & Fabbri, 2013; McFeeters et al., 2013; Ibrahim et al., 2014; Evers et al., 2015; Hendrickx, Mateus & Buffetaut, 2016). Since no synapomorphies of either Carcharodontosauridae or Spinosauridae (and other tetanuran clades) are present in OLPH 025, it is provisionally considered distinct from these taxa.

Most of the features present in the Palermo specimen are shared by ceratosaurid ceratosaurians (e.g., Madsen & Welles, 2000), a clade reported in the Aptian-Albian of South America (Rauhut, 2004) and possibly North Africa (Fanti et al., 2014). Nevertheless, the “ceratosaurid-like” features in OLPH 025 (e.g., distally reclined head, low lesser trochanter placed distally) are symplesiomorphies shared by most non-tetanuran neotheropods. Furthermore, the Palermo specimen apparently lacks the distinct trochanteric shelf present in *Ceratosaurus* (Madsen & Welles, 2000). Among non-tetanuran theropods, OLPH 025 is comparable in overall morphology to the femora of Abelisauridae (e.g., Carrano, 2007; Carrano & Sampson, 2008), as both show a distally reclined head, non-prominent trochanteric shelf, distally placed lesser trochanter of stout, alariform shape, a stocky shaft with the fourth trochanter placed proximally, and rugose muscular insertion areas (e.g., Carrano, 2007). Since the latter group is the only known Late Cretaceous clade of large-bodied non-tetanuran theropods (Carrano & Sampson, 2008) and abelisaurid material is already known from the KKCA (Russell, 1996; Mahler, 2005; D’Orazi Porchetti et al., 2011), we consider it most parsimonious to refer OLPH 025 to Abelisauridae.

### Body size estimation of OLPH 025

Although incompletely preserved, the distal end of OLPH 025 provides information on the minimal mediolateral diameter of the femoral shaft, which we estimate as no less than 115 mm. The same diameter in a 1018 mm long femur of the large abelisaurid *Carnotaurus* measures 95 mm (Carrano, 2006), which may indicate a 1200 mm long femur for the Moroccan individual, comparable to the adult femora of cf. *Bahariasaurus*, *Carcharodontosaurus*, and *Tyrannosaurus* (Carrano, 2007). A length of 1041 mm results using the only known femur of *Xenotarsosaurus* as reference (Juarez-Valieri, Porfiri & Calvo, 2011, table 1). Nevertheless, other abelisaurids show hindlimb proportions stockier than those of *Carnotaurus* and *Xenotarsosaurus* (e.g., *Majungasaurus*, see Carrano, 2007; *Ekrixinatosaurus*, Juarez-Valieri, Porfiri & Calvo, 2011). Therefore, using the gracile-limbed taxa as reference may overestimate the actual length of the Moroccan bone if the latter pertained to the robust morphotype. In particular, the shaft diameter of OLPH 025 is approximately the same as that reported for the type femur of *Ekrixinatosaurus novasi* (shaft diameter, 115 mm; total length, 776 mm), a taxon considered among the most massive abelisauroids by Juarez-Valieri, Porfiri & Calvo (2011). Based on a large sample of theropod femora known from both total length and mediolateral diameter of shaft, we estimate the minimal total length of OLPH 025 as 924 mm (data from Carrano (2006), *N* = 55, *r*^2^ = 0.97). Therefore, we consider a value between 776 and 924 mm as the most conservative estimate for the total length of this Moroccan femur. Using the equation in Christiansen & Farina (2004) to infer total body mass from femur length, a value up to 1850 kg is suggested for this individual, making it among the largest ceratosaurians ever found.

### Palaeoecological implications

The presence in the KKCA of one of the largest known specimens of Abelisauridae confirms that this clade had reached its largest known body size no later than the early Cenomanian (Smith et al., 2010; Juarez-Valieri, Porfiri & Calvo, 2011), and that large-bodied abelisaurids co-existed with giant carcharodontosaurids and spinosaurids in North Africa (Russell, 1996; Sereno, Wilson & Conrad, 2004; Brusatte & Sereno, 2007; Cau, Dalla Vecchia & Fabbri, 2013). Unfortunately, the majority of theropod-bearing localities from North Africa lacks detailed information on the geological context of the dinosaurian material (McGowan & Dyke, 2009; Cavin et al., 2010; Fanti et al., 2014). In absence of detailed stratigraphic, taphonomic, and palaeoecological data, it is unclear whether these large-bodied theropod lineages were sympatric and ecologically overlapping or, on the contrary, each group was constrained to a distinct environmental context, with their co-occurrence in the same depositional setting being mainly due to taphonomic factors (see Hone, Xu & Wang, 2010; Fanti et al., 2014; Hendrickx, Mateus & Buffetaut, 2016). The co-occurrence of giant carcharodontosaurids and large abelisaurids in the KKCA recalls the faunal composition of the Candeleros Formation (Neuquén Basin, Argentina), where both *Giganotosaurus* and *Ekrixinatosaurus* are reported (Juarez-Valieri, Porfiri & Calvo, 2011). In this regards, the Moroccan and Niger assemblages are more similar to the Aptian-Cenomanian faunas from South America (see Novas et al., 2013, and reference therein) than the Cenomanian fauna from Egypt, where no abelisaurids are known (Stromer, 1931; Stromer, 1934; Carrano & Sampson, 2008; Sereno & Brusatte, 2008). On the contrary, the KKCA recalls the Baharjie fauna in the presence of large-bodied and gracile-limbed ceratosaurians (bahariasaurids), the latter unknown from Niger and South America. Among non-theropod dinosaurs, both the Candeleros Formation and the KKCA include rebbachisaurid and basal titanosaurian sauropods (Russell, 1996; Calvo, Rubilar-Rogers & Moreno, 2004): on the contrary, rebbachisaurids appear absent from both Niger and Egypt, whereas titanosaurians are reported in Egypt (Stromer, 1931; Smith et al., 2001). Given the small number of collected individuals belonging to the aforementioned clades, the differences among these faunal assemblages may be artifacts due to sampling bias. Nevertheless, it is worth noting that spinosaurids, abundantly recorded in the KKCA and other African assemblages (Russell, 1996; Dal Sasso et al., 2005; Hone, Xu & Wang, 2010; Ibrahim et al., 2014), are currently absent from the Candeleros Formation (Juarez-Valieri, Porfiri & Calvo, 2011). We therefore consider this faunal difference among the large theropods from the KKCA and the Candeleros Formation as not biased by collecting or taphonomic factors. A possible explanation of the anomalous distribution of spinosaurids, when compared to the other mentioned saurischians, is provided by the theropod record in the Ain El Guettar Formation (Albian of Tunisia, Fanti et al., 2014). In the Ain El Guettar Formation, an abelisaurid-carcharodontosaurid association dominates the lower Chenini Member, characterized by wadi-like channels and arid alluvial plain deposits, whereas spinosaurids dominate the upper Oum ed Diab Member, characterized by estuarine and embayment deposits (Fanti et al., 2014). Assuming that this stratigraphic (and, inferred, ecological and environmental) partition between the large-bodied theropods also characterized other “mid-Cretaceous” associations from Africa and South America, we conclude that spinosaurids were ecologically and environmentally segregated to other large-bodied theropods (Hone, Xu & Wang, 2010). This hypothesis is supported by the morphological specializations of spinosaurids (and, in particular, spinosaurines; Amiot et al., 2010; Ibrahim et al., 2014; Hendrickx, Mateus & Buffetaut, 2016) that suggest a mode of life distinct from that of other theropods. As discussed by Cavin et al. (2010), the KKCA includes at least two distinct formations (the Ifezouane Formation and the overlying Aoufous Formation), with the vast majority of the dinosaurian remains recovered without detailed taphonomic information and often with ambiguous stratigraphic placement. Sereno et al. (1996), distinguished between a lower and upper units of their “Kem Kem beds,” but it is unclear how these two units fit the Ifezouane and Aoufous formations of Cavin et al. (2010) and Hendrickx, Mateus & Buffetaut (2016, supplemental material). Therefore, we conclude that the reported co-occurrence of spinosaurids with abelisaurids and carcharodontosaurids in the KKCA may reflect the lack of stratigraphic resolution in a heterogeneous sample recovered from multiple units rather than a genuine evidence of sympatry and ecological overlap between these theropods.

## Conclusions

The taxonomy and inclusiveness of the theropod clades from the “middle” Cretaceous of North Africa is complex and problematic. Since Stromer (1931) and Stromer (1934) the minimum number of taxa recovered from these fossil associations has been considered controversial, in particular due to the fragmentary nature of most of the specimens found. Stromer himself (1934) was aware of this as one of the main problems in North African dinosaur palaeontology. Several factors, both biological and geological, may bias the taxonomic composition of the North African theropod faunas. Most North African units are poorly constrained stratigraphically (see Cavin et al., 2010; Fanti et al., 2014), thus preventing detailed correlations between the various localities. For example, the age of the KKCA has been alternatively placed between the Aptian and the Cenomanian (Russell, 1996; Cavin et al., 2010), and both number of and relationships among the units represented by that assemblage remain controversial (Sereno et al., 1996; Cavin et al., 2010). The temporal extent of these assemblages is uncertain, possibly spanning several million years (Cavin et al., 2010). Therefore, the application of biological (neontological) “rules,” based on ecological models and data from modern ecosystems (in order to constrain the number of carnivorous taxa included in a fossil assemblage) is often not adequately justified or not testable. This is particularly problematic for fossil assemblages, like the KKCA, that lack present-day analogues and where an unusually unbalanced ecological web has been suggested (e.g., Läng et al., 2013). Since the co-occurrence in the same North African theropod associations of distinct species belonging to the same clade has been documented (e.g., spinosaurids, Fanti et al., 2014; Hendrickx, Mateus & Buffetaut, 2016; carcharodontosaurids, Cau, Dalla Vecchia & Fabbri, 2012; Cau, Dalla Vecchia & Fabbri, 2013), the referral of all isolated elements of one lineage to a single species cannot be justified. Furthermore, the referral of isolated and non-overlapping material to the same species is a phylogenetic hypothesis itself that needs to be explicitly tested by numerical analyses. In absence of positive evidence supporting the referral of such material to a particular species, the inclusion of non-overlapping elements into a single taxon may led to the creation of a potential chimera, with unpredictable effects on the phylogenetic and palaeoecological interpretation of these faunas.

We have described the fragmentary femur of a large-bodied theropod from the “Kem Kem Compound Assemblage” of Morocco. The specimen lacks tetanuran synapomorphies and is referred to Abelisauridae as it shares the overall morphology of the femora of ceratosaurians and the stocky robust proportions of some Late Cretaceous abelisaurids (e.g., *Ekrixinatosaurus*, *Majungasaurus*, Carrano, 2007; Juarez-Valieri, Porfiri & Calvo, 2011). The large size of the preserved femur suggests an individual comparable in body size with the type specimens of *Carnotaurus sastrei* and *Ekrixinatosaurus novasi*, both estimated to reach 9 meters in length and approaching two tons in body mass (Juarez-Valieri, Porfiri & Calvo, 2011). This discovery further supports that abelisaurids had evolved their largest size no later than the “mid-Cretaceous” (Smith et al., 2010; Juarez-Valieri, Porfiri & Calvo, 2011) and that abelisaurids and carcharodontosaurids co-existed and ecologically overlapped in both North Africa and South America during the Aptian-Turonian. Based on comparison with other “middle Cretaceous” units (Juarez-Valieri, Porfiri & Calvo, 2011; Fanti et al., 2014), we suggest that the co-occurrence of spinosaurids and other large theropods (abelisaurids and carcharodontosaurids) in the KKCA may be mainly an artefact due to poor stratigraphic resolution rather than genuine evidence of  ecological and environmental overlap. Given the convergent evolution of several craniodental features among abelisaurids and carcharodontosaurids (Lamanna, Martinez & Smith, 2002; Sampson & Witmer, 2007; Carrano & Sampson, 2008; Cau, Dalla Vecchia & Fabbri, 2013), suggesting similar ecological adaptations in these clades, how these apparently competing groups co-existed for at least 30 million years in both Africa and South America remains to be resolved.

## Additional Information and Declarations

### Data Deposition

The following information was supplied regarding data availability: The research in this article did not generate any raw data.
